# DAMPs Released from Proinflammatory Macrophages Induce Inflammation in Cardiomyocytes via Activation of TLR4 and TNFR

**DOI:** 10.3390/ijms232415522

**Published:** 2022-12-08

**Authors:** Carolina Neu, Yvonne Thiele, Fabienne Horr, Christian Beckers, Nadine Frank, Gernot Marx, Lukas Martin, Sandra Kraemer, Elisabeth Zechendorf

**Affiliations:** Department of Intensive and Intermediate Care, University Hospital RWTH Aachen, 52074 Aachen, Germany

**Keywords:** macrophage, cardiomyocyte, inflammation, septic cardiomyopathy

## Abstract

Cardiac dysfunction is a life-threatening complication in sepsis. Upon infection and cardiac stress, the cardiac macrophage population expands. Recruited macrophages exhibit a predominantly proinflammatory phenotype and release danger-associated molecular patterns (DAMPs) that contribute to cardiac dysfunction. However, the underlying pathomechanisms are highly complex and not fully understood. Here, we utilized an indirect macrophage–cardiomyocyte co-culture model to study the effects of proinflammatory macrophages on the activation of different cardiac receptors (TLR3, TLR4, and TNFR) and their role in cardiac inflammation and caspase-3/7 activation. The stimulation of cardiomyocytes with conditioned medium of LPS-stimulated macrophages resulted in elevated IL-6 protein concentrations and relative IL-6 and TNFα mRNA levels. Conditioned medium from LPS-stimulated macrophages also induced NFκB translocation and increased caspase-3/7 activation in cardiomyocytes. Analyzing the role of different cardiac receptors, we found that TLR4 and TNFR inhibition reduces cardiac inflammation and that the inhibition of TNFR prevents NFκB translocation into the nuclei of cardiomyocytes, induced by exposure to conditioned medium of proinflammatory macrophages. Moreover, we demonstrated that TLR3 inhibition reduces macrophage-mediated caspase-3/7 activation. Our results suggest that the immune response of macrophages under inflammatory conditions leads to the release of DAMPs, such as eRNA and cytokines, which in turn induce cardiomyocyte dysfunction. Thus, the data obtained in this study contribute to a better understanding of the pathophysiological mechanisms of cardiac dysfunction.

## 1. Introduction

Sepsis is a dysregulated host immune response to infection accompanied by severe organ dysfunction that often leads to multi-organ failure [1]. The impairment of cardiac function in terms of ventricular dilation and diminished contractility, referred to as septic cardiomyopathy, is one of the most frequent and most severe health complications in patients with sepsis and is associated with a significant 20–50% increase in mortality [2,3].

The most common trigger of sepsis is lipopolysaccharide (LPS), a major component of the outer membrane of Gram-negative bacteria belonging to the group of pathogen-associated molecular patterns (PAMPs) [4]. Upon infection, innate immune cells, including neutrophils, macrophages, dendritic cells, natural killer cells, and T cells, are key mediators not only in the initial host response, but also in the arising dysregulated immune response causing inflammation [5]. Particularly, macrophages play crucial roles in the regulation of cardiac dysfunction, since cardiac stress is clearly associated with an expanded cardiac macrophage population [6,7]. In addition to the local proliferation of cardiac resident macrophages, circulating monocytes are recruited and differentiated into macrophages [7]. Due to their predominantly proinflammatory phenotype, these recruited macrophages release danger-associated molecular patterns (DAMPs) that contribute to cardiac dysfunction [8].

Once PAMPs or DAMPs are recognized by pattern recognition receptors, including toll-like receptors (TLRs) expressed on the cell surface of cardiomyocytes, intracellular signal cascades induce the activation and translocation of nuclear factor κB (NFκB) and the subsequent release of inflammatory cytokines such as tumor necrosis factor α (TNFα) and interleukin-6 (IL-6) [9,10]. These cytokines, in turn, induce the release of additional inflammatory factors that act on cardiomyocyte homeostasis [11]. For example, TNFα elicits altered Ca^2+^ handling and cell death via activation of the TNF receptor (TNFR) [12].

Inflammatory processes can also be mediated by extracellular RNA (eRNA) via cardiac TLR3 receptor [13]. eRNA belongs to the group of DAMPs and is released from necrotic cells in response to tissue injury in sepsis. Ribonuclease (RNase) 1, an endogenous host-defense peptide secreted from distinct immune cells, can modulate the levels of circulating eRNA by catalytic cleavage [14]. In recent years, the protective effect of RNase 1 treatment has been revealed in various diseases, such as in preserving the blood–brain barrier integrity during the early period following traumatic brain injury [15]. Furthermore, we demonstrated in a previous study that the administration of RNase 1 attenuates cardiac apoptosis and septic cardiac dysfunction in vivo [16].

The release of DAMPs such as eRNA can also occur through macrophage-derived extracellular vesicles (EVs), resulting in therapeutic or pathological roles in cardiac dysfunction [17,18,19]. EVs are nano-sized entities released from almost any cell encapsulating intracellular components such as RNA, proteins, lipids, or metabolites depending on the microenvironment of the donor cell. Cell–cell communication via the EV-mediated transport of such bioactive molecules has a pivotal role in numerous physiological and pathophysiological processes, such as inflammation [7,20]. Although increased numbers of circulating EVs during sepsis have been observed in various studies, their direct impact on cardiomyocytes remains mostly unknown [21,22,23].

To mimic the crosstalk between macrophages and cardiomyocytes, different co-culture models are commonly utilized [24,25,26]. However, due to the fact that cardiomyocytes are directly or indirectly exposed to conditioned medium of proinflammatory macrophages containing cytokines, growth factors, hormones, eRNA, and EVs, the underlying pathomechanisms are highly complex and require further studies [17,27].

Hence, we aimed to study the involvement of specific signaling pathways in cardiac inflammation and apoptosis induced by proinflammatory macrophages. For this purpose, we utilized an indirect co-culture model where cardiomyocytes were challenged with conditioned medium of proinflammatory macrophages. Specifically, the roles of different cardiac receptors, namely TLR3, TLR4, and TNFR, were investigated.

## 2. Results

### 2.1. LPS-Stimulated Macrophages Induce a Strong Cytokine Response in Cardiomyocytes

**Figure 1:**
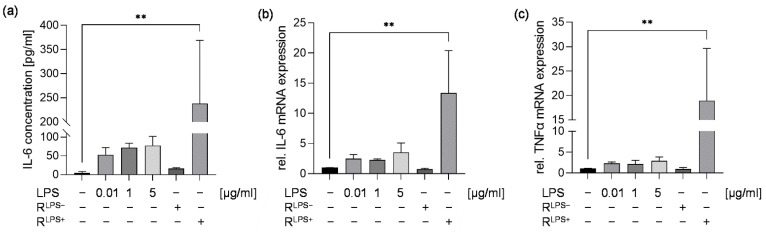
Treatment of cardiomyocytes with conditioned medium of LPS-stimulated macrophages results in an elevated IL-6 concentration and relative IL-6 and TNFα mRNA expression. Cardiomyocytes exposed to either 0.01, 1, or 5 µg/mL LPS or conditioned medium of macrophages stimulated in the presence (R^LPS+^) or absence (R^LPS−^) of 10 ng/mL LPS for 6 h compared with unstimulated cells were analyzed regarding (**a**) IL-6 protein concentrations utilizing ELISA, (**b**) relative IL-6 and (**c**) TNFα mRNA expression measured by quantitative real-time PCR. Data are presented as a column bar graph as the mean ± SD (all n = 3). One-way ANOVA followed by Tukey’s test was used for multiple comparisons. ** *p* < 0.01; LPS = lipopolysaccharide; IL-6 = interleukin-6; TNFα = tumor necrosis factor alpha.

In our study, we initially analyzed the cytokine response of cardiomyocytes after exposure to either 0.01, 1, or 5 µg/mL LPS or conditioned medium of macrophages stimulated in the presence (R^LPS+^) or absence (R^LPS−^) of 10 ng/mL LPS for 6 h. Unstimulated cells were referred to as negative control. Regardless of the concentration, no significant alteration in IL-6 protein concentration and relative IL-6 and TNFα mRNA levels were measured in cardiomyocytes directly stimulated with LPS compared with unstimulated cells. While R^LPS−^ had no effect on the IL-6 protein concentration in cardiomyocyte supernatants and relative IL-6 and TNFα mRNA levels, we found significantly elevated IL-6 protein concentrations and relative IL-6 and TNFα mRNA levels in the supernatants of cardiomyocytes challenged with R^LPS+^ compared with unstimulated cells (*p* < 0.01, Figure 1).

### 2.2. TLR3 Inhibition Reduces Cardiac Caspase-3/7 Activation without Attenuating Cardiac Inflammation and Cardiac Apoptosis Induced by LPS-Stimulated Macrophages

**Figure 2:**
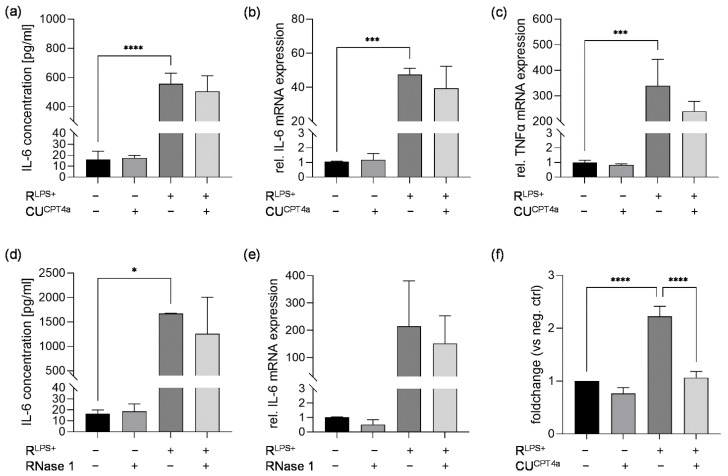
The effects of TLR3 inhibition or RNase 1 treatment on cardiomyocytes exposed to conditioned medium of LPS-stimulated macrophages in terms of cardiac inflammation and/or caspase-3/7 activation. Cardiomyocytes exposed to conditioned medium of LPS-stimulated macrophages (R^LPS+^) in the presence or absence of 40 µM TLR3 inhibitor (CU^CPT4a^) for 6 h compared with unstimulated cells and cells stimulated with the TLR3 inhibitor CU^CPT4a^ were analyzed regarding (**a**) IL-6 protein concentrations quantified by ELISA as well as relative mRNA expression of (**b**) IL-6 and (**c**) TNFα measured by quantitative real-time PCR. (**d**) IL-6 protein concentration and (**e**) IL-6 mRNA expression of cardiomyocytes exposed to conditioned medium of LPS-stimulated macrophages (R^LPS+^) in the presence or absence of 2.8 U/mL RNase 1 for 6 h compared with unstimulated cells and cells stimulated with RNase 1. (**f**) Relative caspase-3/7 activation of cardiomyocytes exposed to conditioned medium of LPS-stimulated macrophages (R^LPS+^) in the presence or absence of 40 µM TLR3 inhibitor (CU^CPT4a^) for 24 h compared with unstimulated cells and cells challenged with the TLR3 inhibitor CU^CPT4a^ using Caspase Glo Assay. Data are presented as column bar graphs as the mean ± SD (all n = 3). One-way ANOVA followed by Tukey’s test was used for multiple comparisons. * *p* < 0.05; *** *p* < 0.001; **** *p* < 0.0001; LPS = lipopolysaccharide; TLR3 = toll-like receptor 3; IL-6 = interleukin-6; TNFα = tumor necrosis factor alpha; RNase 1 = ribonuclease 1.

In an earlier study, we demonstrated that serum eRNA levels are significantly increased in septic patients compared with healthy volunteers. In vivo, we found that eRNA induced significantly increased caspase-3 activation in murine cardiomyocytes (HL-1 cells) compared with unstimulated cells [16]. Therefore, we investigated whether inflammation in cardiomyocytes exposed to R^LPS+^ for 6 h is caused by eRNA binding to TLR3. Cardiomyocytes were challenged with R^LPS+^ in the presence or absence of the TLR3 inhibitor CU^CPT4a^. Interestingly, secreted IL-6 protein concentration and relative IL-6 and TNFα mRNA levels induced by exposure to R^LPS+^ were not significantly altered by TLR3 inhibition (Figure 2a–c). To confirm these results, cardiomyocytes were simultaneously exposed to R^LPS+^ and the eRNA-degrading enzyme RNase 1 for 6 h. Cardiomyocyte stimulation with R^LPS+^ in the presence of RNase 1 had no significant effect on IL-6 protein concentration and mRNA expression compared to cells treated with R^LPS+^ in the absence of RNase 1 (Figure 2d,e). Additionally, we analyzed the effect of TLR3 inhibition on cardiac caspase-3/7 activation. Compared with unstimulated cells, cardiomyocytes exposed to R^LPS+^ for 24 h showed significantly increased caspase-3/7 activation (Figure 2f). Treatment with the TLR3 inhibitor significantly attenuated the increase in caspase-3/7 activation in cardiomyocytes challenged with R^LPS+^ (*p* ≤ 0.0001, Figure 2f).

**Figure 3:**
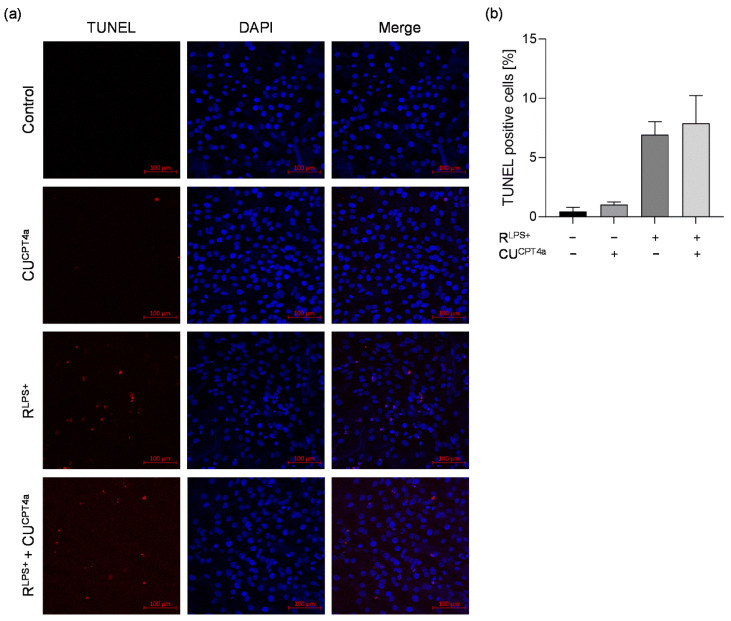
Treatment of cardiomyocytes with conditioned medium of LPS-stimulated macrophages results in increased cardiac apoptosis via a TLR3-independent pathway. Cardiomyocytes exposed to conditioned medium of LPS-stimulated macrophages in the presence (R^LPS+^ + CU^CPT4a^) or absence (R^LPS+^) of 40 µM TLR3 inhibitor CU^CPT4a^ for 24 h compared with unstimulated cells (control) and 40 µM TLR3 inhibitor CU^CPT4a^ (CU^CPT4a^) were analyzed using TUNEL labeling (n = 2). (**a**) Apoptotic cells were labeled with TUNEL (red) and the nuclei of cardiomyocytes were stained with DAPI (blue). Scale bars: 100 μm. (**b**) Quantification of TUNEL fluorescence. Data are presented as column bar graph as the mean ± SD. LPS = lipopolysaccharide; TLR3 = toll-like receptor 3.

After demonstrating that TLR3 inhibition effectively diminishes R^LPS+^-induced cardiac caspase-3/7 activation, we aimed to analyze the effect of TLR3 inhibition on cardiac apoptosis using TUNEL labeling after the stimulation of cardiomyocytes with R^LPS+^ for 24 h (Figure 3a). An increased number of 6.9% TUNEL-positive cells was observed for R^LPS+^-treated cardiomyocytes compared with unstimulated cardiomyocytes (control) that had <1% TUNEL-positive cells. Contrary to its effect on cardiac caspase-3/7 activation, cardiomyocyte exposure to R^LPS+^ in the presence of the TLR3 inhibitor did not show a marked effect on the amount of cardiac TUNEL-positive cells compared with R^LPS+^ (Figure 3b). These results suggest that caspase-3/7 activation is mediated via TLR3, while TLR3 inhibition does not affect the number of TUNEL-positive cells.

### 2.3. TLR4 Inhibition Reduces Cardiac Inflammation Induced by LPS-Stimulated Macrophages

**Figure 4:**
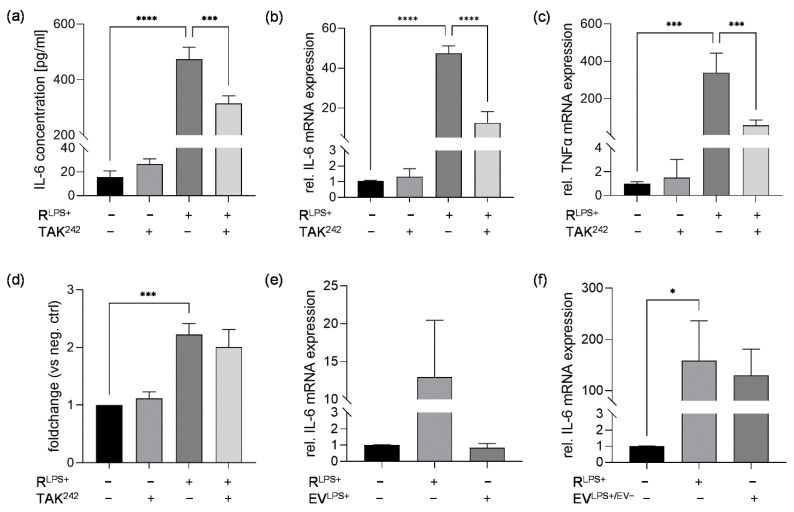
The effects of TLR4 inhibition on cardiomyocytes exposed to conditioned of LPS-stimulated macrophages and cardiomyocyte exposure to either isolated EVs generated from conditioned medium of LPS-stimulated macrophages or EV-depleted conditioned medium of LPS-stimulated macrophages in terms of cardiac inflammation and/or caspase-3/7 activation. Cardiomyocytes exposed to conditioned medium of LPS-stimulated macrophages (R^LPS+^) in the presence or absence of 20 µM TLR4 inhibitor (TAK^242^) for 6 h compared with unstimulated cells and TLR4 inhibitor were analyzed (n = 3). (**a**) IL-6 protein concentrations were measured utilizing ELISA. Relative mRNA expression of (**b**) IL-6 and (**c**) TNFα were determined by quantitative real-time PCR. (**d**) Relative casapase-3/7 activation was assayed using Caspase Glo Assay. Relative IL-6 mRNA expressions of cardiomyocytes stimulated with either (**e**) isolated EVs generated from conditioned medium of LPS-stimulated macrophages (EV^LPS+^, n = 2) or (**f**) EV-depleted conditioned medium of LPS-stimulated macrophages (R^LPS+/EV−^, n = 3) are presented. Data are presented as column bar graphs as the mean ± SD. One-way ANOVA followed by Tukey’s test was used for multiple comparisons. * *p* < 0.05; *** *p* < 0.001; **** *p* < 0.0001; LPS = lipopolysaccharide; TLR4 = toll-like receptor 4; IL-6 = interleukin-6; TNFα = tumor necrosis factor alpha; EVs = extracellular vesicles.

Since cardiac TLR4 activation is known to play a crucial role in the regulation of cardiac dysfunction during sepsis, we examined the effect of TLR4 inhibition on macrophage–cardiomyocyte crosstalk in terms of cardiac inflammation. Again, significantly elevated IL-6 protein concentrations were measured in the supernatants of cardiomyocytes exposed to R^LPS+^ for 6 h. Exposure of cardiomyocytes to R^LPS+^ and a TLR4 inhibitor (TAK-242) resulted in a significant decrease in IL-6 concentration compared to R^LPS+^-stimulated cells (*p* < 0.001, Figure 4a). Compared with R^LPS+^ stimulation, TLR4 inhibition also resulted in significantly decreased mRNA expression of proinflammatory IL-6 (*p* < 0.0001, Figure 4b) and TNFα (*p* < 0.001, Figure 4c). Additionally, R^LPS+^-induced cardiac caspase-3/7 activation was analyzed after 24 h regarding the effect of TLR4 inhibition. Compared with unstimulated cells, cardiomyocytes exposed to R^LPS+^ showed significantly increased caspase-3/7 activation. Treatment with TLR4 inhibitor, however, had no significant effect on caspase-3/7 activation in cardiomyocytes challenged with R^LPS+^ (Figure 4d).

As M1 macrophage-derived EVs are activators of NFκB translocation via cardiac TLR4, we next aimed to investigate whether EVs contained in the conditioned medium of LPS-stimulated macrophages are responsible for the observed TLR4-mediated R^LPS+^-induced cardiac inflammation [28]. Therefore, cardiomyocytes were stimulated with either isolated EVs generated from conditioned medium of LPS-stimulated macrophages (EV^LPS+^) or EV-depleted conditioned medium of LPS-conditioned macrophages (R^LPS+/EV−^). The LPS stimulation period of RAW 264.7 cells was prolonged to 24 h. Consistently, cardiomyocyte exposure to R^LPS+^ for 6 h resulted in elevated relative IL-6 mRNA expression. Interestingly, stimulation of cardiomyocytes with 2.53 × 10^8^ to 4.49 × 10^8^ particles/mL EV^LPS+^ (equivalent to the stimulation volume used in the other experiments) did not alter the relative IL-6 mRNA expression compared to unstimulated cells (Figure 4e). In line with this, the exposure of cardiomyocytes to either R^LPS+/EV−^ or R^LPS+^ resulted in similar relative IL-6 mRNA expression (Figure 4f). We therefore conclude that R^LPS+^-induced cardiac inflammation is induced by a macrophage-released stimulus other than EVs.

### 2.4. TNFR Inhibition Attenuates Cardiac Inflammation and NFκB Translocation Induced by LPS-Stimulated Macrophages

**Figure 5:**
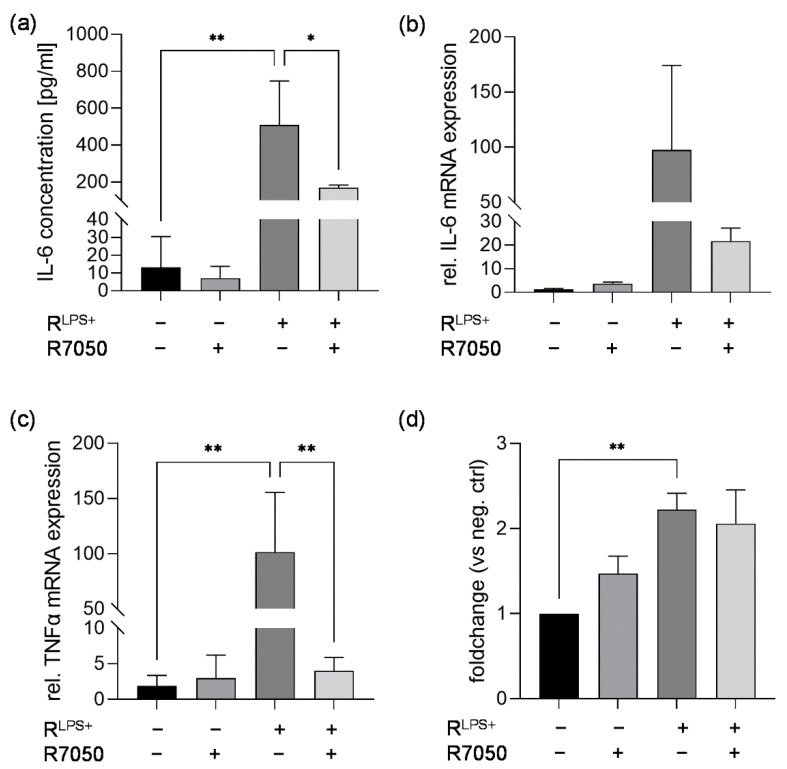
TNFR inhibition reduces cardiac inflammation but not caspase-3/7 activation in cardiomyocytes exposed to conditioned medium of LPS-stimulated macrophages. Cardiomyocytes exposed to conditioned medium of LPS-stimulated macrophages (R^LPS+^) in the presence or absence of 30 µM TNFR inhibitor (R7050) for 6 h compared with unstimulated cells and TNFR inhibitor were analyzed regarding (**a**) secreted IL-6 protein concentrations utilizing ELISA. Relative mRNA expression of (**b**) IL-6 and (**c**) TNFα were determined by quantitative real-time PCR. (**d**) Relative casapase-3/7 activation was assayed using Caspase Glo Assay. Data are presented as column bar graph as the mean ± SD (all n = 3). One-way ANOVA followed by Tukey’s test was used for multiple comparisons. * *p* < 0.05; ** *p* < 0.01; LPS = lipopolysaccharide; TNFR = tumor necrosis factor receptor; IL-6 = interleukin-6; TNFα = tumor necrosis factor alpha.

Besides RNA and extracellular vesicles, proinflammatory cytokines such as TNFα are secreted from proinflammatory macrophages [29]. Since TNFα is associated with exacerbating myocardial dysfunction, we aimed to analyze the role of TNFα signaling via its receptor TNFR in cardiac inflammation and caspase-3/7 activation induced by exposure to R^LPS+^ [12]. Therefore, cardiomyocytes were exposed to R^LPS+^ in the presence or absence of TNFR inhibitor (R7050) for 6 h. Again, significantly elevated IL-6 protein concentrations were quantified measured in the supernatants of cardiomyocytes exposed to R^LPS+^ for 6 h (*p* < 0.01, Figure 5a). The inhibition of TNFR significantly reduced the IL-6 concentration in the cell supernatants compared to R^LPS+^-stimulated cells (*p* < 0.05, Figure 5a). Moreover, the relative IL-6 mRNA expression was increased in cardiomyocytes exposed to R^LPS+^ compared with unstimulated cells. Treatment with R^LPS+^ in the presence of TNFR inhibitor resulted in the decreased relative mRNA expression of IL-6 compared to treatment with R^LPS+^ (Figure 5b). Relative TNFα expression was also significantly increased in cardiomyocytes exposed to R^LPS^ compared with unstimulated cells (*p* < 0.01, Figure 5c). Interestingly, treatment with R^LPS+^ and TNFR inhibitor significantly attenuated relative TNFα mRNA expression to nearly baseline expression levels (*p* < 0.01, Figure 5c). Additionally, R^LPS+^-induced cardiac caspase-3/7 induction was analyzed after 24 h regarding the effect of TNFR inhibition. As expected, cardiomyocytes exposed to R^LPS+^ showed significantly increased caspase-3/7 activation compared to unstimulated cells, while exposure to R^LPS+^ and the TNFR inhibitor had no significant effect on caspase-3/7 activation compared to cardiomyocytes challenged with R^LPS+^ (Figure 5d). These results indicate that R^LPS+^-induced cardiac inflammation, but not caspase-3/7 activation, is mainly induced through binding and activation of TNFR by secreted TNFα.

**Figure 6:**
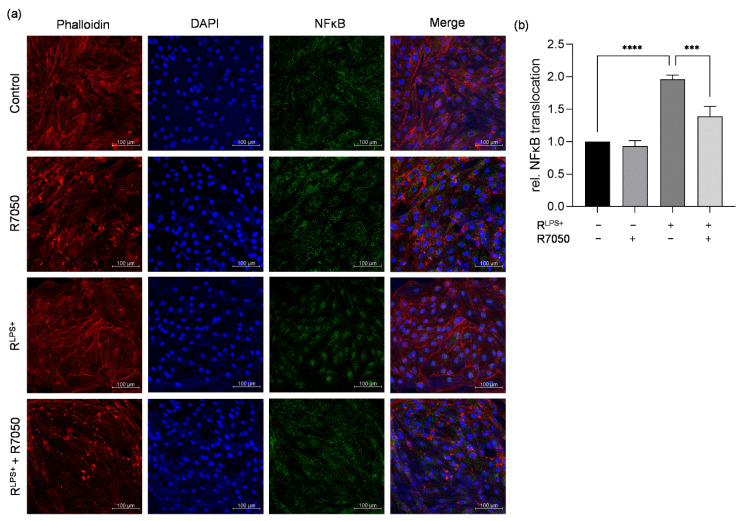
TNFR inhibition diminishes NFκB translocation in cardiomyocytes exposed to conditioned medium of LPS-stimulated macrophages. (**a**) Cardiomyocytes exposed to conditioned medium of LPS-stimulated macrophages (R^LPS+^) for 30 min in the presence (R^LPS+^ + R7050) or absence of TNFR inhibitor (R7050) were stained with phalloidin-TRITC, DAPI, and anti-NFκB and compared with unstimulated cells (n = 3). Phalloidin-TRITC was used to stain the cytoskeleton (red), and nuclei of the cardiomyocytes were stained with DAPI (blue). The green fluorescence indicates whether NFκB is located in the cytoskeleton or is translocated into the nucleus. Scale bars: 100 µm. (**b**) Quantification of NFκB translocation. One-way ANOVA followed by Tukey’s test was used for multiple comparisons. Data are presented as column bar graph as the mean ± SD. *** *p* < 0.001; **** *p* < 0.0001; LPS = lipopolysaccharide; TNFR = tumor necrosis factor receptor; NFκB = nuclear factor kappa-light-chain-enhancer of activated B-cells.

Increased production of proinflammatory cytokines is commonly initiated by the canonical activation and translocation of NFκB [30]. Therefore, we conducted immunofluorescence microscopy to examine whether NFκB is translocated into the nuclei of cardiomyocytes exposed to conditioned medium of LPS-stimulated macrophages for 30 min and whether this can be prevented by TNFR inhibition (Figure 6a). A significantly increased relative NFκB translocation was detected in cardiomyocytes exposed to R^LPS+^ compared with unstimulated cells (*p* < 0.0001, Figure 6b). After exposure of cardiomyocytes to R^LPS+^ in the presence of TNFR inhibitor, a significantly decreased relative NFκB translocation was observed compared to R^LPS+^ (*p* < 0.001, Figure 6b), indicating that the R^LPS+^-induced activation of NFκB is mediated by TNFα signaling via TNFR.

## 3. Discussion

Cardiac dysfunction is a life-threatening complication in sepsis associated with increased mortality and prolonged hospital length of stay [31,32]. Since causal therapeutics are still not available, there is an urgent need to improve current understanding of the pathogenesis of septic cardiomyopathy [3]. In future studies, clinical characteristics as well as the underlying biochemical mechanisms need to be thoroughly investigated to identify potential therapeutic targets.

As early as 1989, Salari and colleagues demonstrated that cardiac dysfunction associated with endotoxemia or septic shock is caused by factors released from endotoxin-activated macrophages [33]. However, in vitro studies addressing septic cardiomyopathy have been commonly based on LPS stimulation of cardiomyocytes. Frequently observed limitations are the use of unphysiologically high LPS concentrations ranging from 1 to 10 µg/mL and the use of undifferentiated cardiomyoblasts [34,35,36,37,38]. Such studies also neglected the involvement of immune cells, such as macrophages, as part of the immune response to infection. More recently, the interaction between macrophages and cardiomyocytes has been the focus of research. In fact, macrophage-mediated cardiac inflammation has been demonstrated in various indirect or direct co-culture models mimicking the macrophage–cardiomyocyte crosstalk [24]. In a direct comparison, we found that differentiated cardiomyoblasts directly exposed to 0.01, 1, or 5 µg/mL LPS did not show a proinflammatory response. In contrast, IL-6 protein concentrations were significantly increased in cardiomyocytes challenged with conditioned medium of macrophages exposed to 0.01 µg/mL LPS, suggesting that the macrophage–cardiomyocyte model is more valid. Importantly, conditioned medium of unstimulated macrophages did not affect cardiomyocyte cytokine responses, implying that there are no unspecific interspecies cross-reactions between murine macrophages and rat cardiomyocytes.

Since cardiomyocytes are challenged with conditioned medium of macrophages, a heterogeneous mixture of cytokines, growth factors, hormones, eRNA, EVs, and DAMPs, the underlying pathophysiology is highly complex and not fully understood. To gain further mechanistic insight, we aimed to study various signaling pathways through the inhibition of different cardiac receptors (TLR4, TLR3, and TNFR).

In the context of septic cardiomyopathy, activation of TLR4 is considered a crucial step contributing to cardiac dysfunction. In fact, we found that TLR4 inhibition significantly attenuated cardiac inflammation induced by conditioned medium of proinflammatory macrophages. However, baseline levels of the inflammatory markers were not reached by TLR4 inhibition, suggesting that macrophage-induced cardiac inflammation is only partially mediated via TLR4. Based on our initial findings demonstrating that directly stimulating cardiomyocytes with 0.01 µg/mL LPS does not induce a proinflammatory response, we concluded that LPS, potentially remaining in the conditioned medium, does not contribute to TLR4-mediated cardiac inflammation. Thus, we hypothesized that DAMPs released from macrophages may activate cardiac TLR4. For example, cellular stress provokes the release of high mobility group box protein 1 (HMGB1) from various cells into the extracellular space [39]. Upon release, HMGB1 is a prototypical DAMP that plays a decisive role in inflammatory processes and immunosuppression during sepsis and other inflammatory diseases [40]. Recently, Wang and colleagues demonstrated a time-dependent increase in HMGB1 protein expression in RAW 264.7 cells after exposure to LPS and that HMGB1 is released as the cargo of EVs [41]. Additionally, it was previously shown that M1 macrophage-derived EVs induce NFκB translocation via TLR4 in neonatal rat ventricular myocytes [28]. Hence, we hypothesized that EVs released into conditioned medium of LPS-challenged macrophages may induce TLR4-mediated cardiac inflammation. Against our expectations, exposure of cardiomyocytes to isolated EVs generated from conditioned medium of LPS-stimulated macrophages had no effect on cardiac inflammation in terms of IL-6 protein concentrations. Furthermore, we analyzed EV-depleted conditioned medium of LPS-stimulated macrophages in cardiomyocyte stimulation, which resulted in significantly elevated IL-6 protein concentrations, thus supporting our finding that proinflammatory macrophage-derived EVs do not mediate cardiac inflammation. Other TLR4-binding DAMPs that trigger inflammatory responses in sepsis are circulating histones released from activated immune cells such as neutrophils and macrophages [42]. Previous studies have demonstrated that LPS-challenged macrophages release both circulating and EV-bound histones and that direct stimulation with histones has a dose-dependent cytotoxic effect on isolated rat cardiomyocytes [43,44]. Moreover, DAMPs released from inflammatory cardiomyocytes may contribute to TLR4-mediated cardiac inflammation. However, although we could reveal the involvement of cardiac TLR4 in macrophage-mediated inflammation, the present study fails to identify the trigger of TLR4 activation.

In 2011, He and colleagues reported that macrophages undergo necrosis when TLR4 is activated by LPS [45]. Based on these findings, we hypothesized that eRNA released from necrotic macrophages may activate intracellular cardiac TLR3 and downstream signaling pathways important in macrophage–cardiomyocytes crosstalk during cardiac dysfunction. Interestingly, we observed that TLR3 inhibition had no effect on macrophage-mediated cardiac cytokine response, indicating that signaling of eRNA via TLR3 does not contribute to cardiac inflammation. Our results are consistent with earlier studies demonstrating that TLR3^−/−^ mice and wild-type mice had similar cytokine responses after ischemia–reperfusion [13]. Appropriately, the treatment of cardiomyocytes with eRNA-degrading peptide RNase 1 did not affect the proinflammatory cytokine response induced by conditioned medium of proinflammatory macrophages.

It was previously demonstrated that undifferentiated H9c2 cardiomyoblasts co-cultured with LPS-treated RAW 264.7 macrophages show increased caspase-3 protein expression [25]. Co-culturing with bone marrow-derived macrophages in the presence of LPS also exhibited increased caspase-3 expression [26]. Additionally, our data provide evidence that macrophage-induced caspase-3/7 activation is mediated via TLR3 but not TLR4. Specifically, we demonstrated that TLR3 inhibition significantly attenuates R^LPS+^ induced caspase-3/7 activation. Further, we assumed that induction of the proapoptotic cascade via TLR3 results in cardiac apoptosis.

Although we observed indications of cardiac apoptosis in terms of an increased number of TUNEL-positive cells, TLR3 inhibition had no effect on R^LPS+^-induced cardiac apoptosis. Given the strong macrophage-mediated inflammatory response in cardiomyocytes, the proportion of TUNEL-positive cells was generally low, suggesting that caspase-3/7 activation does not result in cardiac apoptosis.

In 1989, Virca and colleagues demonstrated that stimulation of RAW 264.7 macrophages with LPS induces elevated steady state levels of TNFα mRNA [46]. Since TNFα is one of the most potent proinflammatory cytokines, we hypothesized that soluble TNFα in the conditioned medium of LPS-stimulated macrophages may activate cardiac TNFR, contributing to cardiac dysfunction. Hence, we aimed to investigate the role of TNFR signaling in cardiac inflammation. Indeed, the inhibition of cardiac TNFR attenuates the proinflammatory cytokine response induced by the conditioned medium of LPS-stimulated macrophages.

TNFR-dependent inflammation is associated with NFκB activation [47]. Specifically, a previous study reported that NFκB is activated in adult human cardiomyocytes exposed to human recombinant TNFα for 30 min [48]. In line with this study, nuclear NFκB was detected in cardiomyocytes exposed to conditioned medium of proinflammatory macrophages for 30 min. In cardiomyocytes treated in the presence of TNFR inhibitor, NFκB was localized in the cytoplasm. Quantification of our immunofluorescence images revealed that TNFR inhibition effectively prevents NFκB translocation, indicating that TNFR activation is a crucial step in macrophage-mediated NFκB-dependent cardiac inflammation. Moreover, these results support our previous finding that cardiac inflammation induced by proinflammatory macrophages is mediated not only via TLR4, but also via TNFR.

Earlier, we suggested that stimulation with conditioned medium of LPS-stimulated macrophages may lead to necroptosis in cardiomyocytes, which may contribute to an increased amount (>6.9%) of cardiac cell death. Necroptosis, also referred to as programmed necrosis, is induced through defective polyubiquitination of receptor-interacting protein kinase (RIPK) 1 and phosphorylation of RIPK3, which forms a necrosome with mixed lineage kinase domain-like (MLKL) pseudokinase [49,50]. Although we demonstrated the involvement of TNFR in cardiac inflammation, soluble TNFα can, via activated TNFR, provoke the activation of other signaling pathways contributing to cell survival, apoptosis, and necroptosis. Regarding the tight connection between apoptosis, pyroptosis, and necroptosis as well as their ability to crossregulate each other, additional studies are needed to investigate whether proinflammatory macrophages induce cardiac necroptosis [51].

The present study has several limitations. (a) Since we demonstrated the involvement of cardiac TLR4 in macrophage-mediated inflammation but failed to identify the trigger of TLR4 activation, future investigations are needed to address the role of circulating histones potentially released into conditioned medium of proinflammatory macrophages in cardiac inflammation. (b) Regarding cell death, we solely studied macrophage-mediated cardiac apoptosis, neglecting the potential presence of other forms of cell death, such as pyroptosis and necroptosis, as well as their cross-regulatory functions. (c) In this study, we successfully established an indirect in vitro co-culture model to study macrophage-mediated cardiac dysfunction. Septic cardiomyopathy is a very complex disease which is characterized by reduced left ventricular (LV) contractility and dilation. In addition to cardiomyocyte dysfunction, pathophysiological processes also include endothelial, metabolic, and immune response abnormalities. We are aware that our crosstalk model only focuses on a small part of this complex disease and that further studies and more physiological models, e.g., primary cardiomyocytes, induced pluripotent stem cell derived cardiomyocytes, or animal models, are needed to further investigate the contribution of crosstalk to septic cardiomyopathy.

## 4. Materials and Methods

*Cell Culture*. RAW 264.7 cells (ATCC, Wesel, Germany), a murine macrophage cell line, and H9c2 cells, a rat heart myoblast cell line, were cultured in Gibco Dulbecco’s Modified Eagle’s Medium (ThermoFisher, Waltham, MA, USA) supplemented with 10% fetal bovine serum (FBS, Merck, Darmstadt, Germany) and 1% penicillin/streptomycin (ThermoFisher) under a humidified atmosphere of 5% CO_2_ at 37 °C. All experiments were conducted in differentiated H9c2 cells (ATCC). Cardiac differentiation of H9c2 cells was performed using culture media containing 1% FBS and adding 100 nM all-trans retinoic acid (RA, Merck) daily in the dark for 5 days [52]. Differentiated H9c2 cells are referred to as cardiomyocytes.

*Cell Stimulation*. In order to generate conditioned medium, RAW macrophages were stimulated with 10 ng/mL LPS (Merck) or left untreated. After 6 h incubation, the supernatants were harvested, centrifuged at 4 °C and 300 *g* for 5 min, and stored at −80 °C. H9c2 cardiomyocytes were stimulated with conditioned RAW medium in the presence or absence of 20 µM TAK 242 (TLR4 inhibitor, Merck), 40 µM CU CPT 4a (TLR3 inhibitor, Bio-Techne GmbH, Wiesbaden-Nordenstadt, Germany), 30 µM R7050 (TNFR inhibitor, Selleck Chemicals LLC, Houston, TX, USA), 2.8 U/mL ribonuclease 1 (Merck), and 2.53 × 10^8^ to 4.49 × 10^8^ particles/mL isolated EVs generated from conditioned RAW medium or EV-free conditioned RAW medium for 6 or 24 h. Unstimulated cells were used as a negative control.

*Relative mRNA Expression*. Total RNA from H9c2 cardiomyocytes was isolated using TRIzol reagent, as previously described [16,53]. Afterwards, cDNA was synthesized using a Maxima H Minus First Strand cDNA Synthesis kit (ThermoFisher). In order to analyze the relative mRNA expression of IL-6, TNFα in a quantitative real-time PCR (StepOnePlus Real-Time PCR System, ThermoFisher) Power SYBR Green PCR Master mix (Applied Biosystems, CA, USA) was utilized. The mRNA expression levels were then normalized against S7, and the relative mRNA levels were calculated using the 2^−ΔΔCt^ method. The following primers were used for amplification: S7: for: 5′-AACACGTAGTCTTCATTGCTC-3′; rev: 5′-AAAGTTTCGACCTTGT-GCTC-3′; IL-6: for: 5′-TCTCCGCAAGAGACTTCCA-3′; rev: 5′-CAATCAGAATTGCCA-TTGCAC-3′; TNFα: for: 5′-CTCAGCCTCTTCTCATTCCT-3′; rev: 5′-CTTGGTGGTTTGC-TACGAC-3.

*ELISA*. The levels of IL-6 in the supernatants of H9c2 cardiomyocytes were determined using a rat IL-6 DuoSet ELISA kit (Bio-Techne) with an assay range from 9.4 to 600 pg/mL. The assay was performed according to the manufacturer. Briefly, 96-well assay plates were coated with a capture antibody diluted in PBS overnight. Afterwards, the plate was blocked and incubated with reagent diluent (1% BSA (Merck) in PBS) for at least 1 h. Next, the standard and samples were added to the plate for 2 h, followed by incubation with a detection antibody for 2 h. The plate was then incubated with streptavidin-HRP for 20 min in the dark. After each described step, the plate was washed three times with a washing buffer (0.05% Tween^®^ 20 (Merck) in PBS). Finally, the plate was incubated with Pierce^TM^ TMB substrate kit (ThermoFisher). The reaction was stopped using 2 N H_2_SO_4_. The optical density was measured at 450 and 570 nm as reference wavelengths using a microplate reader (Infinity 200, Tecan, Männedorf, Switzerland). According to the manufacturer, 0.7% cross-reactivity with recombinant mouse IL-6 is known. However, no IL-6 was detected in the conditioned RAW medium.

*Caspase-3 activity*. In order to analyze caspase-3 activity, 6 x 10^3^ H9c2 cells per well were seeded and differentiated in 96-well white plates. H9c2 cardiomyocytes were stimulated for 24 h, as described above. Afterwards, caspase-3 activity was analyzed by adding Caspase-Glo 3/7 Assay reagent (Promega, Walldorf, Germany) in a 1:1 ratio followed by an incubation period of 30 min at room temperature. The luminescence was detected using a microplate reader (Infinity 200, Tecan).

*TdT-Mediated dUTP-Biotin Nick End (TUNEL) Labeling*. H9c2 cells were grown and differentiated in 12-well plates on acid-washed coverslips. H9c2 cardiomyocytes were fixed with 4% paraformaldehyde (Merck) for 1 h at room temperature after stimulation for 6 h as described above. After washing, cells were permeabilized with 0.1% Triton X-100 (Merck) and 0.1% sodium citrate (AppliChem, Darmstadt, Germany) for 2 min on ice. Labeling of the cells was performed using the In Situ Cell Death Detection Kit TMR red (Roche, Mannheim, Germany) as previously described [53]. Briefly, nuclei of apoptotic H9c2 cells were stained by incubation with the TUNEL reaction mixture for 1 h at 37 °C in the dark. During this reaction, apoptosis-induced DNA strand breaks are labeled with modified nucleotides. The washing step was repeated and followed by incubation with DAPI (Merck, diluted 1:1000 in PBS) for 10 min at room temperature to stain nuclei of all H9c2 cells. Coverslips were washed twice and covered with Vectashield HardSet Antifade Mounting Medium (Biozol, Eching, Germany). A LSM 710 confocal microscope (Zeiss, Oberkochen, Germany) and ImageJ software (NIH, Bethesda, MD, USA) were used to detect apoptotic cells.

*Immunofluorescence of NFκB*. To analyze the translocation of NFκB into the nucleus, H9c2 cells were seeded in 12-well plates on acid-washed coverslips. After differentiation, as described above, H9c2 cells were exposed to conditioned RAW medium in the presence or absence of 20 µM TAK 242, 40 µM CU CPT 4a, or 30 µM R7050 for 30 min. Afterwards, cells were fixed with 4% paraformaldehyde for 15 min at room temperature. After two washing cycles using PBS, cells were permeabilized and blocked with 5% normal goat serum (Bio-Techne) and 0.3% Triton X-100 in PBS for 1 h at room temperature. Next, the H9c2 cells were incubated with a primary antibody directed against NFκB p65 (#4764, Cell Signaling, Danvers, MA, USA), diluted 1:100 in antibody dilution buffer (1% BSA and 0.3% Triton X-100 in PBS) overnight at 4 °C. After six washing cycles, the cells were simultaneously incubated with the Alexa Fluor 488 anti-rabbit secondary antibody (ThermoFisher), diluted 1:400 in antibody dilution buffer and phalloidin-TRITC (Merck), a fluorescent cytoskeleton stain, and diluted 1:4000 in antibody dilution buffer for 2 h at room temperature in the dark. Again, six washing cycles were performed, and the nuclei of the cells were stained using DAPI, diluted 1:1000 in PBS, for 10 min at room temperature. Coverslips were washed twice and covered with Vectashield HardSet Antifade Mounting Medium. Immunofluorescence was detected using a LSM 710 confocal microscope (Zeiss) and quantified using ImageJ software.

*EV Purification from Cell Culture Supernatants*. For EV isolation, RAW 264.7 stimulation was prolonged to 24 h. Afterwards, RAW 264.7 cell culture supernatants of three 15 Ø dishes were pooled for each group, respectively, after stimulation in the presence or absence of 10 ng/mL LPS. EVs were isolated, as described previously [54]. Briefly, differential centrifugation steps were performed at 4 °C for 5 min at 300× *g*, then 10 min at 2000× *g*, and finally 45 min at 12,000× *g*, followed by filtration through a 0.22-µm filter. Vivaspin 20 concentrators (100,000 Dalton cut-off, Satorius AG, Göttingen, Germany) were sterilized with 2 × 15 mL of 70% ethanol and centrifugation for 2 min at 6000× *g*. Next, the concentrators were washed with 15 mL of PBS for 2 min at 6000× *g*. The cell supernatants were concentrated to a final volume of 500 µL. EVs were purified utilizing Exo-spin™ columns (Cell Guidance Systems, Cambridge, United Kingdom) for size exclusion chromatography. The columns were equilibrated with 2 × 10 mL of sterile PBS before 500 µL of concentrated supernatant was applied. In total, 12 fractions with a 500 µL volume were collected in LoBind^®^ tubes (Eppendorf, Hamburg, Germany). Protein concentrations of the potentially EV-enriched Fractions 5–12 were determined using a microbicinchoninic acid (BCA) assay (Bio-Rad, Hercules, CA, USA). EV isolation was confirmed by visualizing particle size and concentration of Fractions 7–9 using nanoparticle tracking analysis. For H9c2 cell stimulation, Fractions 8 and 9 were pooled.

*Preparation of EV-Depleted Cell Supernatants*. Again, RAW 264.7 cells were stimulated for 24 h. In order to remove EVs from the harvested conditioned RAW medium, it was subjected to differential centrifugation as described above. Afterwards, ultracentrifugation of the supernatants was performed for 1 h at 110,000× *g* and 4 °C (Optima XE, Beckman Coulter, Krefeld, Germany). Finally, the pellet was discarded, and the supernatants were pooled and stored at −80 °C.

*Statistical Analysis*. GraphPad Prism 9 (GraphPad Inc., San Diego, CA, USA) was used to perform the statistical analysis and to create the graphs. One-way ANOVA followed by Tukey’s test was used for multiple comparisons with a significance level of *p* < 0.05. Data are presented as mean ± SD for n = 3 (TUNEL staining and the experiments with isolated EVs for n = 2) independent experiments in triplicate.

## 5. Conclusions

**Figure 7:**
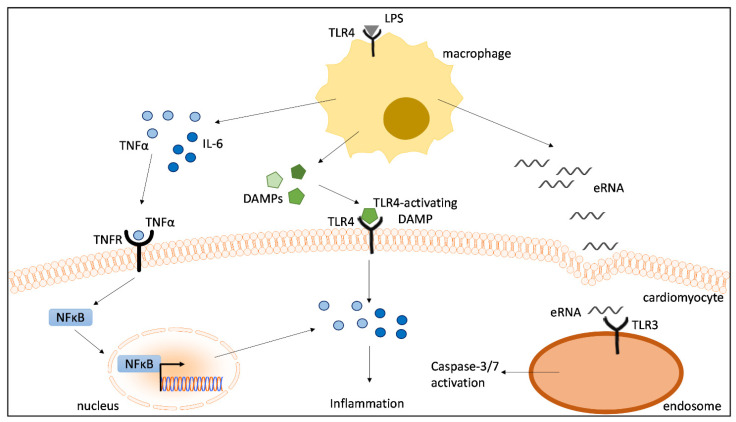
Proinflammatory macrophages induce cardiac inflammation and caspase 3/7 activation. Cytokines, eRNA and other unknown DAMPs released from LPS-stimulated macrophages contribute to cardiac inflammation via the activation of TLR4- and TNFR-dependent NFκB translocation as well as to TLR3-mediated cardiac caspase-3/7 activation. LPS = lipopolysaccharide; TLR = toll-like receptor; TNFR = tumor necrosis factor receptor; NFκB = nuclear factor kappa-light-chain-enhancer of activated B-cells; IL-6 = interleukin-6; TNFα = tumor necrosis factor alpha; eRNA = extracellular RNA.

We established an in vitro macrophage–cardiomyocyte crosstalk model utilizing conditioned medium of LPS-stimulated macrophages to stimulate cardiomyocytes. Compared to simple LPS stimulations of cardiomyocytes, our crosstalk model appears to be more suitable in recreating pathophysiological processes during septic cardiomyopathy. The present study shows that conditioned medium of LPS-stimulated macrophages induces elevated IL-6 and TNFα levels, while direct LPS stimulation has no effect on the cardiac cytokine response. Here, we demonstrated for the first time that (a) TLR4 or TNFR inhibition reduces cardiac inflammation, (b) the inhibition of TNFR effectively prevents NFκB translocation into the nuclei of cardiomyocytes, and (c) TLR3 inhibition suppresses caspase-3/7 activation induced by conditioned medium of proinflammatory macrophages (Figure 7). Generally, our data reveal that the pathophysiological mechanisms in cardiomyocytes are highly complex and substantially affected by the immune response of macrophages. Based on these findings, we propose that DAMPs released from LPS-stimulated macrophages significantly contribute to cardiac inflammation via the activation of TLR4- and TNFR-dependent NFκB translocation, as well as to TLR3-mediated cardiac caspase-3/7 activation (Figure 7). These data contribute significantly to increasing the understanding of macrophage-mediated cardiac dysfunction in sepsis.

## Data Availability

Not applicable.
